# Prevalence and determinants of online-sex use in the German population

**DOI:** 10.1371/journal.pone.0176449

**Published:** 2017-06-19

**Authors:** Manfred E. Beutel, Sebastian Giralt, Klaus Wölfling, Yve Stöbel-Richter, Claudia Subic-Wrana, Iris Reiner, Ana Nanette Tibubos, Elmar Brähler

**Affiliations:** 1 Department of Psychosomatic Medicine and Psychotherapy, University Medical Center, Mainz, Germany; 2 Faculty of Managerial and Cultural Studies, University of Applied Sciences Zittau/ Goerlitz, Goerlitz, Germany; Ariel University, ISRAEL

## Abstract

**Introduction:**

The unlimited access to sexual features in the World Wide Web has raised concerns about excessive and problematic online-sex use. However, little is known about antecedents of internet-sex use of different intensity. Based on a representative German sample of 2,522 participants between the ages of 14 and 97 years, the aims of the present study were (1) to determine the prevalence rates of online-sex users with the short version (ISST_GSV_) of the Internet Sex Screening Test and (2) to associate online-sex use with anxious vs. avoidant partner attachment patterns and “Big Five” personality traits as potential antecedents.

**Results:**

The ISST is a brief, one-dimensional and reliable measure of online-sex activities (r_tt_ = .69). Overall, 14.7% of respondents reported occasional and 4.2% intensive online-sex use. In multivariate analysis, online-sex use was significantly positively associated with male sex, younger age, unemployment and an anxious partner attachment pattern and negatively with conscientiousness and agreeableness.

**Conclusions:**

Arousal and satisfaction by virtual enactment of sexual phantasies may be attractive for anxiously attached persons who find it difficult to commit to a real life relationship due to fear of rejection or low self-esteem. More knowledge about the individual antecedents of intensive online-sex use may also be helpful for the development of consultation and treatment strategies for excessive and addictive online-sex use.

## Introduction

The internet offers an almost unlimited variety of sexual materials, commodities, activities and information serving all sexual orientations. All kinds of pornography can be viewed, downloaded or collected, accounting for the most widespread form of cybersex which has found increasing acceptance in the general population. These and multiple other interactive online options like sexual messaging, chats, dating, engaging in sexual activities on webcam, etc. are easily accessible and affordable at low or no cost, can be consumed anonymously [1] and provide escape from stress and hassles [2]. Of particular concern has been the instantaneous gratification offered by the internet which may account for rapid conditioning of online addictive behaviour [3; 4].

Complementary and infrequent users of solitary or partnered online sexual arousing material have described a positive, albeit small impact on their sex life and life in general [5]. The internet has also provided a comparatively “safe” space for sexual exploration by otherwise marginalized populations of homosexual, transgendered or disabled populations. So far, research has focused on negative repercussions, when online sexual activities have led to sexual harassment, or became excessive dominating the users’ lives [6].

Already in 1998, Young [7] has proposed online-sex addiction as a subcategory of internet addiction, along with online-gaming and gambling, etc. However, online-gaming has become the only kind of internet addiction with a sufficient data base to be specified in the DSM-5. To date, a consistent definition of online-sex addiction is still lacking and its conceptualization has remained controversial [8; 9]. Dhuffar and Griffiths have defined online-sex addiction as “a maladaptive pattern of sexual behaviour, leading to clinically significant impairment or distress” (p. 8; [6]). Similar to other types of online addiction, additional criteria include tolerance (requiring an increasing amount or intensity of sexual behaviour), withdrawal, loss of control, mood regulation by online sexual activity and its persistent use despite negative repercussions. Online-sex use is mostly accompanied by masturbation.

Indeed, excessive sexual activities on the internet have been associated with manifold negative consequences like decreasing partnership quality, vocational achievement, personal distress and high rates of comorbid mental disorders (see for a review [10]). Studies showed associations between higher online pornography consumption and low self-esteem as well as depressive symptoms [11; 12; 13]. Moreover, problematic use of online-sex can take a downward course, facilitating deviant behaviour like watching or downloading illegal, e.g. paedophilic material [14]. Prevalence rates of internet-sex addiction are still unclear [6]. Previous epidemiologic studies have not provided reliable prevalence data on cybersex addiction, since most have been carried out on the Internet, based on English-speaking populations. The estimates ranged from 1% to 8.6% ([15] vs. [16]) of the Internet users being potentially affected by cybersex addiction. While the majority of these users were male, cybersex use, however, has recently also been observed and studied in females [4]. Explanation models for a higher prevalence in men are scarce. One assumption is that women generally find mainstream pornography less arousing than men; since men generally begin masturbating at an earlier age, they may use pornography more intensively than women [17].

One of the first questionnaires for self-assessment of online-sex behaviour, the Internet Sex Screening Test (ISST; sexhelp.com), has been introduced by Delmonico in 1997 [18]. Delmonico and Miller [19] reported the data of 14,656 persons who had completed the questionnaire online. Cronbach’s alpha ranged between .51 to .86 for the seven subscales online sexual compulsivity, sexual behaviour- social, sexual behaviour- isolated, online sexual spending, interest in online sexual material, non-home computer use for online sexual behaviour and accessing illegal sexual material [19]. The so-called “sexual compulsives” spent more time online on sexual purposes, and they also scored significantly higher on all subscales of the ISST. Although higher scores may indicate problematic online sexual behaviour, it is important to bear in mind, that there is no valid cut-off score. Other more recent questionnaires have assessed different constructs. As a measure of excessive sexual behaviour, the Hypersexual Behavior Inventory [20] assesses the dimensions of loss of control, coping and consequences. The Problematic Pornography Use Scale is a reliable and recently validated scale with 12 items covering distress and functional problems, excessive use, control difficulties and use for escape/ avoidance of negative emotions [21]. Specifically for assessing cybersex, the internet addiction test was modified as a 12 item version [3].

Little is known about antecedents of various sexual online activities. As we could show in a representative population survey of German internet users [22], men reported online-sex use four times more often (21.5%) than women (4.7%); young men (below 34 years) had the highest preference. The IPACE (Interaction of Person-Affect-Cognition-Execution) model [23] recently proposed that personality, social cognition, psychopathology and bio-psychological constitution interact with specific motives for using certain internet applications which may evolve into a specific internet use disorder over a series of steps. With regard to online hypersexual behaviour, internet-related cognitive biases are of crucial importance for the maintenance of the disorder [23]. Findings regarding personality dispositions, e.g. the dimensions of the NEO-FFI Inventory (extraversion, openness, etc.; [24]) have been sparse and contradictory. While one study found an association between sensation seeking and sexual exposure in adolescents [25], the other did not find an association [26].

Attachment patterns are known to have a pervasive impact on the quality of intimate and sexual relationships [27]. Kor et al. [21] found a significant, but weak association between avoidance (*r* = .23), respectively anxiety (*r* = .26) as dimensions of insecure attachment and problematic pornography viewing in a sample with a large proportion of students.

Therefore the aims of the present article were (1) to identify the prevalence rates of online-sex use in the German population by a short version of the translated ISST [18] and (2) to determine the associations of the intensity of internet-sex use (none endorsed, occasional, intensive) to basic dimensions of (anxious vs. avoidant) partner attachment and the “Big Five” personality traits. We hypothesized that (1) participants with an avoidant or a fearful-clinging attachment use internet-sex offers more intensively. (2) We expected more intensive online-sex use in extraverted and open participants and less in agreeable and conscientious participants.

## Materials and methods

### Participants

The present study is based on a representative survey of the German population (as confirmed by ADM-sample). Data were collected by USUMA (Unabhängiger Service für Umfragen, Methoden und Analysen; Berlin) between June 01 and July 14, 2011. The research institute follows the general German guidelines for the assessment of minors (e.g. Arbeitskreis Deutscher Markt- und Sozialforschungsinstitute e.V.). All participants were notified orally of the research background of the study, the voluntary nature and the right of a revocation of their own participation at any time. In addition to an accompanying official letter from the client about the research project, a data privacy statement was passed which assured the strict confidentiality of all information in the questionnaire and informed exactly about the handling of personal data. Thus, it was warranted that the address determined had only been detected for the allocation of the data set during the oral on-site survey (and possibly a subsequent verification of the correct implementation for quality assurance of the data).

Out of a total of 2,555 participants (1,341 women; 1,214 men) between the ages of 14 and 97 years who were recruited in 258 sample points, representing East and West Germany, *N* = 2,522 filled out the ISST and were used as our study sample. The majority (80.4%) lived in the Western states of Germany. Households and target persons living in the households were selected by random-route procedure. Participants were interrogated by face-to-face-interviews by trained interviewers in their homes and independently filled out additional questionnaires in the presence of the interviewer. No incentives were offered for study participation. All subjects gave written informed consent and filled in several questionnaires. The procedure was approved by the ethics committee of the University of Leipzig. Fifty-nine percent of the initial sample (4,386 households) were interviewed. This quota matches other representative population samples. The mean age was 49.6 years (*SD* = 18.15). A total of 4.2% of the participants were not of German nationality. Fifty-seven percent lived in a partnership, and 48.6% were married. Eighty-one percent had less than high-school education. The full or part-time employment rate was 46.5% and the unemployment rate 7.0%; 30.7% received pension. More than half of the monthly household incomes were between 750 and 2,000 Euro.

The capacity for insight in adolescents in the age group 14–17 years may be generally assumed. In Germany no separate parental consent is required for participation in this age group. If no legal representative is present at the survey of young people, the information sheet about data protection must be handed out. Any selected target person receives the data protection statement, as this is necessary to fulfil the informed consent regulation.

### Questionnaires

Demographic data included age (≥14 years), sex, marital and employment status.

The German short-version (ISST_GSV_) was derived from the Internet Sex Screening Test, a 25 item measure of online sexual behaviour, which was tested on 5,005 males and 1,083 females from a sexual helpline [19]. As we could only include few items into our survey, we selected one item per scale and two items for sexual compulsivity. As the factor loadings were reported in the Spanish validation study by Arnal [28], we selected the items from the Spanish trial. Items were translated independently from English to German and back except for the two single scale items which were not reported in the English version in full wording and which were therefore translated back and forth from the Spanish version. The items included were (scales in parentheses):”I believe I am an internet-sex addict”; “I have made promises to myself to stop using the Internet for sexual purposes” (Online Sexual Compulsivity); “I have a sexualized username or nickname that I use on the Internet”; (Sexual Behavior- Social);”I have searched for sexual material through an Internet search tool” (Sexual Behavior- Isolated);”I have purchased sexual products online” (Online Sexual Spending); the single scale items were “I have accessed sexual sites from other computers besides my home”; “I have run across illegal sexual material while on the Internet”. Items are rated “true” or “false” on a dichotomous scale. Considering its brevity, we found an acceptable internal consistency r_tt_ = .69 of the Kuder-Richardson-Formula.

In order to measure attachment we used the German adaptation of the Experiences in Close Relationships Questionnaire [29; 30], a measure of self-assessed partner attachment. High scores on the internally consistent (Cronbach´s alpha = .88/.89) and independent (*r* = .05) subscales (18 items, each) represent the two dimensions of insecure attachment: avoidance of emotional closeness and fearfulness of loss of the attachment figure. Avoidant partner attachment is characterized by a lack of romantic love and low relationship success whereas anxious partner attachment is described by possessive love, fear of loss and low self-esteem [30].

The Big Five Inventory (BFI-10) assesses the five basic and bipolar personality dimensions with two items (one positive and one negative pole) each, to be rated on a five-point scale from 1 = “does not apply at all” to 5 = “applies completely”: extraversion (sociable, energetic, outgoing, talkative, assertive vs. reclusive, quiet), agreeability (trusting, altruistic, cooperative, modest vs. critical, distrustful), conscientiousness (ambitious, organized, dutiful, self-disciplined, reliable vs. neglectful, erratic), neuroticism (even-tempered, stable vs. worrisome, brooding, pessimistic, self-conscious, self-indulgent, vulnerable) and openness (imaginative, inquisitive vs. rigid, conservative). It can be considered valid, as the 5-factor structure could be replicated, and there was a consistent pattern of correlations with the NEO Five-Factor Inventory (NEO-FFI). Mean retest reliability was .73 [31].

Depression was measured with the German version of the Patient Health Questionnaire depression module (PHQ-9; [32]) (Cronbach´s alpha in the present study = .83). The frequency of occurrence of symptoms in the past two weeks was rated from “0 = not at all, 1 = several days, 2 = over half the days, and 3 = nearly every day”. Answers are added to a total score (0 to 27); a score ≥ 10 indicating moderate to severe depressive symptoms [33]. Generalized anxiety was assessed using the GAD-7 (Generalized Anxiety Disorder [GAD]-7 Scale) [34]: “Feeling nervous, anxious or on edge” and “not being able to stop or control worrying”. Subjects answered on the same scales as on the PHQ-9. A sum score ≥ 10 (range 0 to 21) indicated moderate to severe anxiety symptoms, performing well as a screening tool for all anxiety disorders [35]. The internal consistency in the current study was Cronbach´s alpha = .77.

### Statistical analysis

We defined three groups of internet-sex users according to the arithmetic mean of positive answers (i.e. total points; maximum 7 points; *M =* .36; *SD =* .89) given in the ISST_GSV_ and the standard deviation. “Intensive online-sex use” was defined as two standard deviations above the mean (≥3 points). “Occasional online-sex use” was defined as one or two endorsed Items (≥ 1 point; ≤ 2 points). The third group endorsed no item. Additionally, a principal component analysis was performed to corroborate the single factor structure of the scale. The Scree test revealed one main component, explaining 36.1% of variance that can be interpreted as “intensive online-sex use”. The range of the factor loadings was .39 to .74. The discriminatory power of the single items ranged between .24 and .54.

In order to investigate the association of intensive online-sex use with demographic and mental health characteristics, Chi^2^-tests for categorical data were performed. Effect sizes were determined by calculating Cramer’s V. With regard to the link of online-sex use with personality variables, bivariate correlation analyses (Pearson) were performed. Bonferroni correction was calculated to address the problem of multiple comparisons. With correction for seven comparisons, the critical alpha level is set at .007 (.05 divided by 7). Finally, we ran a forward hierarchical stepwise multiple linear regression using the enter method defining the sum score of the ISST_GSV_ as outcome variable in order to identify relevant predictors of intensive online-sex use. The first block included gender, age, marital status, partnership and unemployment as independent variables, the second block comprised the Big Five personality traits as well as anxious and avoidant attachment. Calculations were performed by using SPSS Version 23.

## Results

### Prevalence of online-sex use and corresponding sociodemographic data

A total of 14.7% reported occasional online-sex use endorsing one or two items, 4.2% of the participants endorsed three or more items. As they scored two standard deviations above the mean score of the ISST_GSV_, they were considered intensive users. Table 1 compares the group with no item endorsed to occasional and intensive users.

**Table 1:** Demographic data and mental health characteristics: No item endorsed, occasional and intensive online-sex use (N = 2522).

	No item endorsed (n = 2044)	Occasional use	Intensive use (n = 107)	Sign.
		(n = 371)		
**Prevalence**	81.0%	14.7%	4.2%	
**Age (years)**^**1**^	51.7 (18.0)	40.5 (15.4)	36.99 (13.59)	*χ^2^*(10) = 1730.78***, *Cramer’s V* = .19
14–24 (n = 271)	8.7%	18.6%	22.5%	
25–34 (n = 338)	11.5%	20.8%	23.4%	
35–44 (n = 395)	14.3%	20.5%	24.3%	
45–54 (n = 496)	19.4%	21.0%	20.6%	
55–64 (n = 423)	18.3%	11.6%	5.6%	
≥65 (n = 599)	27.7%	7.5%	0.7%	
**Sex**				*χ^2^*(2) = 109.86***, *Cramer’s V* = .21
Male	43.1%	62.5%	85.0%	
Female	56.9%	37.5%	15.0%	
**Partnership**				*χ^2^*(2) = 18.67 ***, *Cramer’s V* = .09
Yes	58.5%	53.7%	38.3%	
No	41.5%	46.3%	61.7%	
**Marital status**				*χ^2^*(8) = 138.17***; *Cramer’s V* .17
Married _living together_ together	50.8%	43.1%	24.3%	
Married _living separately_	1.7%	1.6%	0.0%	
unmarried	21.9%	40.4%	56.1%	
Divorced	11.8%	11.9%	15.9%	
Widowed	13.8%	3.0%	3.7%	
**Unemployment**				*χ^2^*(2) = 16.54***; *Cramer’s V* = .08
Yes	93.8%	90.3%	85.0%	
No	6.2%	9.7%	15.0%	
**Employment status**				*χ^2^*(8) = 152.52*** *Cramer's V* = .17
Employed	47.5%	58.5%	61.7%	
Student/training	6.3%	16.7%	15.0%	
Unemployed	6.2%	9.7%	15.0%	
Retired	34.8%	12.1%	5.6%	
Household	5.1%	3.0%	2.8%	
Depression (PHQ-9)^1^	3.20 (3.95)	3.29 (3.96)	3.32 (3.95)	*χ^2^*(2) = 3.07, *p* = n.s. *Cramer's V* = .04
Anxiety (GAD-7)^1^	2.44 (3.03)	2.62 (3.10)	2.99 (3.06)	*χ^2^*(2) = 1.77, *p* = n.s. *Cramer's V* = .03

- Note

^1^Means, standard deviations in parentheses; Sex: 1 = male, 2 = female; Unemployment: 0 = no, 1 = yes; Employment status: employed = 1, student = 2; unemployed = 3; retired = 4, household/homemaker = 5; Partnership: 1 = living with partner, 2 = living without partner; Marital status: 1 = married and living together, 2 = married, living separately, 3 = single, 4 = divorced, 5 = widowed; Depression and anxiety mean scores

*** *p* ≤ .001.

Intensive online-sex users were significantly younger than those endorsing no item and occasional users. The frequency of intensive online-sex use was more than six times higher among males than among females; the majority of intensive users were either single or unmarried. Intensive online-sex users were more often unemployed. No differences were found regarding residency in Eastern or Western Germany, urban or rural areas and education.

### Online-sex use according to gender

**Fig 1:**
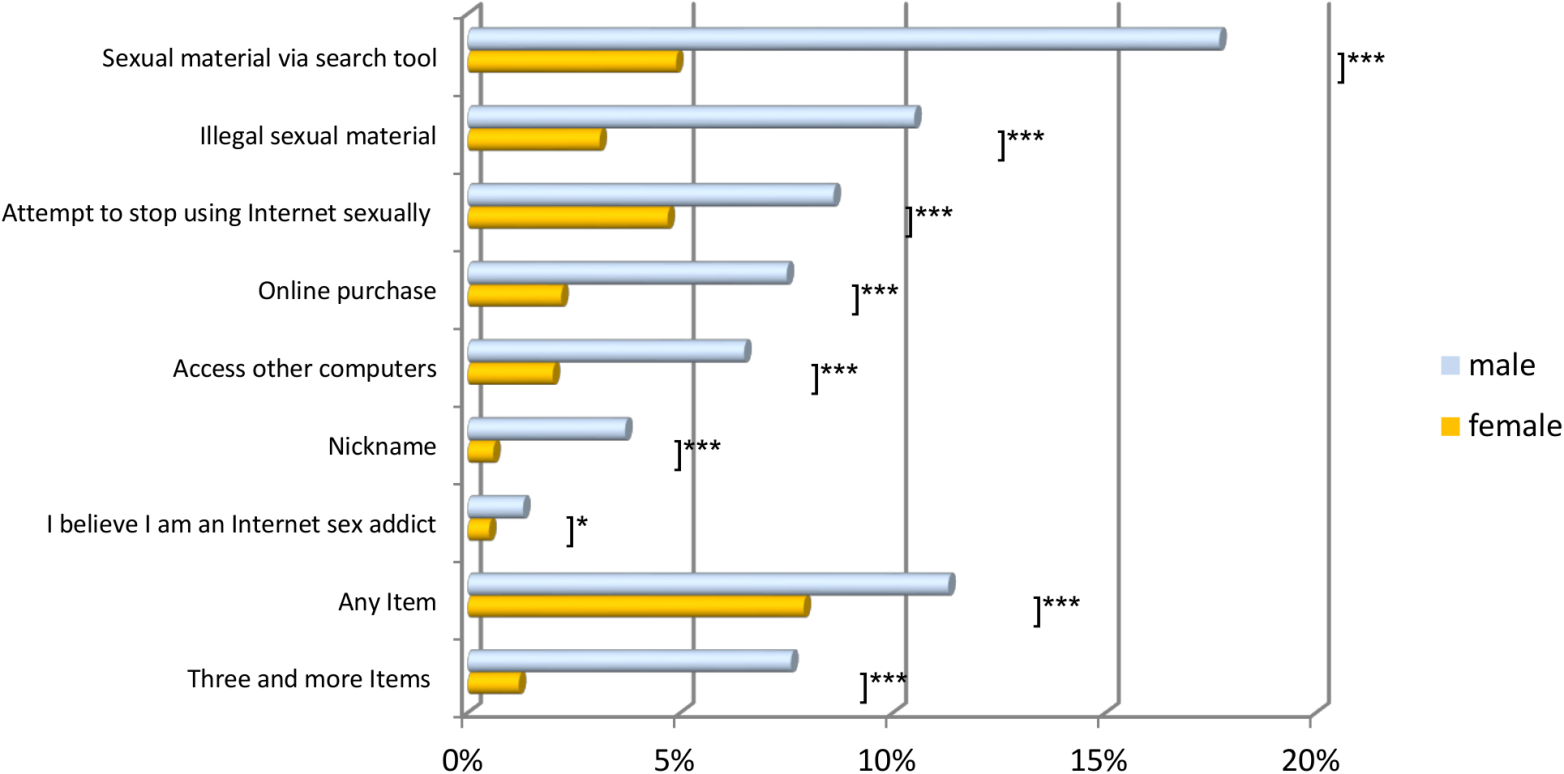
Online-sex use according to gender (N = 2,522). *p ≤ .05; ***p ≤ .001; three and more items indicated intensive sex use.

As Fig 1 shows, men scored significantly higher on every item; intensive use was also more frequent in men. Men showed the same order of preferences as women.

### Personality variables as predictors of online-sex use

**Fig 2:**
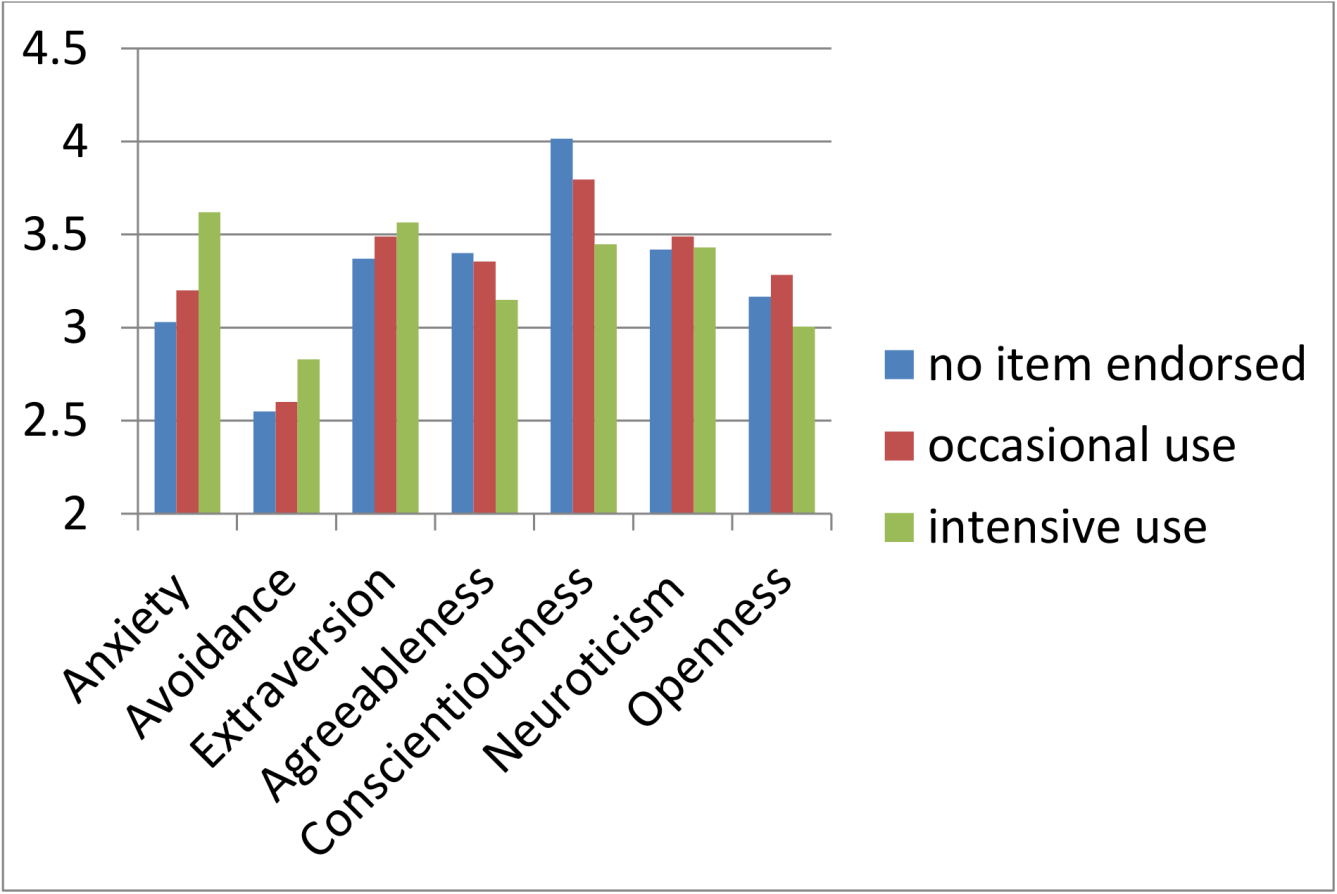
Attachment and personality according to online-sex use. Analysis of variance (ANOVA) by group: N = no item endorsed (N = 1,935); O = occasional use (N = 354); I = intensive use (N = 103); Scheffé-test: a); 1) *F*(2) = 11.79; *p* < .001; 2) *p* < .10; 3) F(2) = 4.65; *p* = .01; 4) *F*(2) = 6.42; *p* = .002, N>O>I; 5) *F*(2) = 34.86; *p* < .001; N>O>I; 6) n.s.; 7) *F*(2) = 4.93; *p* = .007; *** *p* ≤ .001; **p ≤ .01; *p ≤ .05.

Fig 2 shows the associations of different intensities of online-sex use (no item endorsed, 14.7% occasional, 4.2% intensive) with attachment patterns and personality (for bivariate correlations, cf. supplementary table).

Consistent with our predictions, the intensity of internet-sex use increased highly significantly with the degree of anxious attachment. There was only a statistical trend to higher avoidant attachment among intensive users. Also in line with our hypotheses, intensity of internet-sex use increased significantly with extraversion, and decreased with agreeableness and conscientiousness. Openness was lower in intensive and higher in occasional users compared to non-users. There was no association of intensity of internet-sex use with neuroticism. We then tested, whether attachment and personality were predictive of internet-sex use in addition to demographic variables.

Regression analyses revealed that the demographic variables gender, age and unemployment were significant predictors of online-sex use with an explained variance of 11%, *F*(5, 2363) = 58.97; *p* < .001 (see Table 2, block 1). Men, younger and unemployed participants were more likely to use the internet for sexual purposes. When entering the second block of predictors consisting of personality traits, these demographic predictors remained statistically significant. Additionally, anxious attachment, low conscientiousness and low agreeableness turned out to be significant predictors, R2 = .14, F(5, 2363) = 58.97; p < .001 (see Table 2, block 2). S1 Table (Suplementary Table) shows bivariate correlation analyses (Pearson) of online-sex use, partner attachment and personality variables.

**Table 2:** Predictors of online-sex use (N = 2,369).

Predictors	B	SE	Beta	t	Sign.		ΔR^2^
						*Block 1*	.11***
Sex	-.38	.04	-.22	-11.04	.000		
Age	-.01	.00	-.23	-11.26	.000		
Unemployment	.22	.07	.06	3.20	.001		
Partnership	.04	.02	.06	1.86	.064		
Marital status	.04	.06	.02	0.60	.549		
						*Block 2*	.03***
Sex	-.38	.04	-.21	-10.51	.000		
Age	-.01	.00	-.19	-8.89	.000		
Unemployment	.15	.07	.04	2.14	.032		
Partnership	.01	.06	.00	0.12	.902		
Marital status	.04	.02	.06	1.68	.093		
Anxious attachment	.10	.02	.11	5.23	.000		
Avoidant attachment	-.01	.02	-.01	-0.40	.689		
(BFI-10) Conscientiousness	-.15	.02	-.13	-6.07	.000		
(BFI-10) Agreeableness	-.05	.03	-.04	-2.03	.043		
(BFI-10) Openness	.01	.02	.01	0.67	.502		
(BFI-10) Extraversion	.04	.02	.04	1.63	.104		
(BFI-10) Neuroticism	-.04	.03	-.04	-1.67	.095		
						*Overall*	.14***

- Note

***p ≤ .001; Sex: 1 = male, 2 = female; Unemployment: 0 = no, 1 = yes: Partnership: 1 = living with partner, 2 = living without partner; Marital status: 1 = married and living together, 2 = married, living separately, 3 = single, 4 = divorced, 5 = widowed. BFI-10 = Big Five Inventory, 10 item version.

## Discussion

In this large and representative paper and pencil survey of problematic online-sex use in the community, a total of 14.7% of the participants reported consuming online-sex occasionally. In order to define intensive online-sex use, we used a conservative cut-off score (two standard deviations above the mean) and found that a total of 4.2% of the participants used the internet for sexual purposes intensively. This rate was lower compared to previous online studies on excessive use, which may have recruited selected samples of internet users. Yet, reported use according to the ISST_GSV_ items exceeded a previous population survey [36].

The ISST_GSV_ comprises seven dichotomous items and can be administered as a screener for online-sex use. Intensive online-sex use was defined by the endorsement of three or more items. Specific online-sex behaviours differed in frequency. The item “I have searched for sexual material through an Internet search tool” was endorsed by far most often; “I believe I am an internet-sex addict” was the least often endorsed. Other less frequently endorsed items indicated a growing involvement with online-sex behaviour. Search engines pose few restrictions, but it requires some internal pressure and risk taking to log on to another person’s computer (also: workplace) for the purpose of viewing pornographic material.

In line with a recent survey showing more pornography use and masturbation in men compared to women, men reported a much higher rate of online-sex use than women. The reasons for the phenomenon are still to be investigated. Findings on sexual arousal suggested that women tended to be more focused on verbal stimulation [37] and preferred online-sex as mutual exchange to explicit sexual images and thus tended to engage in sexually oriented chats [15, 38–40]. Men seemed to be more visually oriented [36] surfing the World Wide Web more frequently for new pictures and video clips [14, 37]. In univariate analyses, the prevalence of participants with intensive online-sex use increased with each decade until the age span between 35 and 44 years.

The association of intensive online-sex use to anxious partner attachment is an interesting finding. The internet provides unlimited opportunities for enacting all kinds of phantasies in a virtual world, experiencing sexual arousal and satisfaction (e.g. by masturbating). This may be highly attractive for insecurely attached individuals, especially when they find it difficult to commit to a real-life relationship due to fear of rejection or low self-esteem. For anxiously attached participants living in a partnership, online-sex use may provide a compensatory function, boosting self-esteem in a self-governed world of virtual sexual arousal parallel to an offline relationship which may rather induce fears of dependence, rejection or loss. It is not clear, why avoidant attachment is only marginally associated with online-sex use, unlike the findings of Kor [21]. As in the previous study [30], individuals rating themselves as avoidant were less likely to have a partnership. Those individuals may be less interested in sexuality and intimacy due to fear of rejection.

Consistent with the IPACE model [23], personality dispositions also played a role as determinants. As we had expected, online-sex use increased with extraversion, and it decreased with agreeableness and conscientiousness; neuroticism was not associated. Openness was comparatively low in intensive users, but highest in occasional users. A person with an open, imaginative, aesthetically interested, self-aware, unconventional, curious and flexible stance may occasionally use online-sex material, but may be less drawn to an intensive use. We recently found lower conscientiousness, along with higher neuroticism and lower extraversion in patients addicted to internet gaming disorder compared to healthy controls [24]. It is therefore tempting to speculate that different personality dispositions may predispose to different kinds of intensive internet use: While both types of intensive online behaviours are associated with low conscientiousness, extraversion may predispose to online-sex use, while neuroticism may predispose to online-gaming. In multivariate analyses, conscientiousness and agreeableness were predictive of lower online-sex use, both indicating good abilities for social and personal adjustment.

More knowledge about the antecedents of online-sex use may also be helpful to the development of consultation and treatment strategies for addicted online-sex users. Our findings suggest that it may be worthwhile for the clinician to take not only demographic factors (male sex, younger age, partnership, unemployment), but also attachment patterns and personality dispositions into account, e.g. to address fearful and clinging, respectively avoidant partner attachment in the treatment.

Due to the cross-sectional nature of the survey, we cannot draw causal conclusions. I.e. while we surmised that attachment affects online-sex use, it cannot be precluded, that long-term and intensive online-sex use may adversely affect partner attachment. While we differentiated the intensity of online-sex use, we did not differentiate specific activities (e.g. viewing pornography vs. online-dating), nor legal and illegal online activities. We do not know if activities were clandestine, performed by participants themselves or together with their partner. Relationships of internet-sex use and distress, attachment and personality variables were consistent, but small. In future studies it may be necessary to take a closer look at the subgroup with intensive online-sex use, e.g. unemployed, young men.

### Limitations

The brevity of the ISST_GSV_ makes it useful for epidemiological studies, however, as a screening instrument it does not fully differentiate sexual online activities (e.g. use of chats), and it does not specify viewing pornography online. Therefore, we cannot conclude that those who did not endorse at least one item did not use online sex material at all. The dichotomous answering format limits its use for assessing addiction, and it lacks an agreed-upon cut-off score. As we did not specify a time frame for the assessment, the prevalence of intensive online-sex may be overestimated. While we could gain a representative sample, face to face questioning may also induce socially desirable responses in these personal items.

In summary, the present article shows that online-sex use has become widespread in our society; intensive use may be related to attachment difficulties in romantic relationships and also to personality traits. This article also represents the basis for further research within a subject which is yet to emerge. Further research should combine the fully translated German version of the ISST [19] with assessment tools for personality traits and social relationships and skills.

## Supporting information

S1 Table Supplementary table: Bivariate correlation analyses (Pearson) of online-sex use, partner attachment and personality variables (N = 2,522). (DOCX)

## References

[1] Cooper A. Sexuality and the Internet: Surfing into the new millennium. CyberPsychol Behav. 1998;1(2):187–93.

[2] Young KS, Griffin-Shelley E, Cooper A, O'Mara J, Buchanan J. Online infidelity: A new dimension in couple relationships with implications for evaluation and treatment. Sexual Addiction & Compulsivity. 2000;7(1–2):59–74.

[3] Laier C, Pekal J, Brand M. Cybersex addiction in heterosexual female users of Internet pornography can be explained by gratification hypothesis. Cyberpsychol Behav Soc Netw. 2014;17(8):505–11. doi: 10.1089/cyber.2013.0396 25080011 10.1089/cyber.2013.0396

[4] Riemersma J, Sytsma M. A new generation of sexual addiction. Sexual Addiction & Compulsivity. 2013;20(4):306–22.

[5] Shaughnessy K, Byers ES, Clowater SL, Kalinowski A. Self-appraisals of arousal-oriented online sexual activities in university and community samples. Arch Sex Behav. 2014;43(6):1187–97. doi: 10.1007/s10508-013-0115-z 23740466 10.1007/s10508-013-0115-z

[6] Dhuffar MK, Griffiths MD. A systematic review of online sex addiction and clinical treatments using CONSORT evaluation. Curr Add Rep. 2015;2(2):163–74.

[7] Young KS. Internet addiction: The emergence of a new clinical disorder. Cyberpsychol Behav. 1998;1(3):237–44.

[8] Carnes P, Adams KM. Clinical management of sex addiction. New York: Brunner Routledge; 2002.

[9] Kafka MP. The development and evolution of the criteria for a newly proposed diagnosis for DSM-5: Hypersexual disorder. Sexual Addiction & Compulsivity. 2013;20(1–2):19–26.

[10] Griffiths MD. Internet sex addiction: A review of empirical research. Addict Res Theory. 2012;20(2):111–24.

[11] Spenhoff M, Kruger THC, Hartmann U, Kobs J. Hypersexual behaviour in an online sample of males: Associations with personal distress and functional impairment. J Sex Med 2013;10:2996–3005. doi: 10.1111/jsm.12160 23578375 10.1111/jsm.12160

[12] Levert NP. A comparison of Christian and non-Christian males, authoritarianism, and their relationship to internet pornography addiction/compulsion. Sexual Addiction & Compulsivity. 2007;14(2):145–66.

[13] Weaver JB III, Weaver SS, Mays D, Hopkins GL, Kannenberg W, McBride D. Mental‐and Physical‐Health Indicators and Sexually Explicit Media Use Behavior by Adults. J Sex Med. 2011;8(3):764–72. doi: 10.1111/j.1743-6109.2010.02030.x 20946159 10.1111/j.1743-6109.2010.02030.x

[14] Griffiths M. Sex on the Internet: Observations and implications for Internet sex addiction. J Sex Res. 2001;38(4):333–42.

[15] Cooper A, Delmonico DL, Burg R. Cybersex users, abusers, and compulsives: New findings and implications. Sexual Addiction & Compulsivity. 2000;7(1–2):5–29.

[16] Cooper A, Griffin-Shelley E, Delmonico DL, Mathy RM. Online sexual problems: Assessment and predictive variables. Sexual Addiction & Compulsivity. 2001;8(3–4):267–85.

[17] Reid RC, Carpenter BN, Lloyd T. Assessing psychological symptom patterns of patients seeking help for hypersexual behavior. Sex Relat Ther 2009;24:47–63.

[18] Delmonico DL. Internet Sex Screening Test. 1997. Available from: http://www.sexhelp.com/

[19] Delmonico D, Miller J. The Internet Sex Screening Test: A comparison of sexual compulsives versus non-sexual compulsives. Sexual and Relationship Therapy. 2003;18(3):261–76.

[20] Reid RC, Li DS, Gilliland R, Stein JA, Fong T. Reliability, validity, and psychometric development of the Pornography Consumption Inventory in a sample of hypersexual men. J Sex MarTher. 2011;37(5):359–85. 10.1080/0092623X.2011.607047 21961444

[21] Kor A, Zilcha-Mano S, Fogel YA, Mikulincer M, Reid RC, Potenza MN. Psychometric development of the Problematic Pornography Use Scale. Addict Behav. 2014;39(5):861–8. doi: 10.1016/j.addbeh.2014.01.027 24583276 10.1016/j.addbeh.2014.01.027

[22] Beutel ME, Brähler E, Glaesmer H, Kuss DJ, Wölfling K, Müller KW. Regular and problematic leisure-time internet use in the community: results from a German population-based survey. Cyberpsychol Behav Soc Netw. 2011;14: 291–6. doi: 10.1089/cyber.2010.0199 21067277 10.1089/cyber.2010.0199

[23] Brand M, Young KS, Laier C, Wölfling K, Potenza MN. Integrating psychological and neurobiological considerations regarding the development and maintenance of specific Internet-use disorders: An Interaction of Person-Affect-Cognition-Execution (I-PACE) model. Neurosci Biobehav Rev. 2016 8 30;71:252–266. doi: 10.1016/j.neubiorev.2016.08.033 27590829 10.1016/j.neubiorev.2016.08.033

[24] Müller KW, Koch A, Dickenhorst U, Beutel ME, Duven E, Wölfling K. Addressing the question of disorder-specific risk factors of internet addiction: a comparison of personality traits in patients with addictive behaviors and comorbid internet addiction. Biomed Res Int. 2013;2013:546342 doi: 10.1155/2013/546342 23865056 10.1155/2013/546342 PMC3707207

[25] Luder M-T, Pittet I, Berchtold A, Akre C, Michaud P-A, Suris J-C. Associations between online pornography and sexual behaviour among adolescents: Myth or Reality? Arch Sex Behav. 2011; 40:1027–1035. doi: 10.1007/s10508-010-9714-0 21290259 10.1007/s10508-010-9714-0

[26] Brand M, Laier C, Pawlikowski M, Schächtle U, Schöler T, Altstötter-Gleich C. Watching pornographic pictures on the internet: Role of sexual arousal ratings and psychological–psychiatric symptoms for using internet sex sites excessively. Cyberpsychol Behav Soc Netw. 2011 6;14(6):371–7. doi: 10.1089/cyber.2010.0222 21117979 10.1089/cyber.2010.0222

[27] Schimmenti A, Passanisi A, Gervasi AM, Manzella S, Famà FI. Insecure attachment attitudes in the onset of problematic Internet use among late adolescents. Child Psychiatry Hum Dev. 2014;45(5):588–95. doi: 10.1007/s10578-013-0428-0 24338269 10.1007/s10578-013-0428-0

[28] Arnal RB, Llario MDG, Martínez SG, Juliá BG. Propiedades psicométricas de un instrumento de evaluación de la adicción al cibersexo. Psicothema. 2010;22(4):1048–53. 21044551

[29] Brennan KA, Clark CL, Shaver PR. Self-report measurement of adult attachment: an integrative overview. In: JA Simpson, WS Rholes (Eds.), Attachment theory and close relationships. New York: Guilford; 1998. pp. 46–76.

[30] Neumann E, Rohmann E, Bierhoff H-W. Entwicklung und Validierung von Skalen zur Erfassung von Vermeidung und Angst in Partnerschaften. Diagnostica. 2007; 53: 333–47.

[31] Rammstedt B. The 10-item Big Five Inventory: Norm values and investigation of sociodemographic effects based on a German population representative sample. Eur J Psychol Assess. 2007;23(3):193.

[32] Kroenke K, Spitzer RL, Williams JB. The PHQ‐9. J Gen Int Med. 2001;16(9):606–13. 10.1046/j.1525-1497.2001.016009606.x PMC1495268 11556941

[33] Löwe B, Spitzer RL, Gräfe K, Kroenke K, Quenter A, Zipfel S, et al Comparative validity of three screening questionnaires for DSM-IV depressive disorders and physicians’ diagnoses. J Aff Dis. 2004;78(2):131–40. 10.1016/s0165-0327(02)00237-9 14706723

[34] Spitzer RL, Kroenke K, Williams JB, Löwe B. A brief measure for assessing generalized anxiety disorder: the GAD-7. Arch Int Med. 2006;166(10):1092–7. 16717171 10.1001/archinte.166.10.1092

[35] Kroenke K, Spitzer RL, Williams JB, Monahan PO, Löwe B Anxiety disorders in primary care: prevalence, impairment, comorbidity, and detection. Ann Int Med. 2007;146(5):317–25. 17339617 10.7326/0003-4819-146-5-200703060-00004

[36] Richters J, de Visser RO, Badcock PB, Smith AM, Rissel C, Simpson JM, et al Masturbation, paying for sex, and other sexual activities: the Second Australian Study of Health and Relationships. Sexual Health. 2014;11(5):461–71. doi: 10.1071/SH14116 25376999 10.1071/SH14116

[37] Buss DM. Evolutionary psychology: The new science of the mind. Boston: Allyn & Bacon; 1999.

[38] Griffiths M. Sex addiction on the Internet. Janus Head. 2004;7(1):188–217.

[39] Cooper A, Scherer CR, Boies SC, Gordon BL. Sexuality on the Internet: From sexual exploration to pathological expression. Prof Psychol Res Pr. 1999;30(2):154.

[40] Carnes P. Don't call it love: Recovery from sexual addiction. New York: Bantam Books; 1991.

