# Supplementary material for: Prevalence and determinants of online-sex use in the German population
Source: PLoS One. 2017 Jun 19;12(6):e0176449. doi: 10.1371/journal.pone.0176449 (PMC5476251; doi:10.1371/journal.pone.0176449)
Supplement: S1 Table — (DOCX) [file pone.0176449.s001.docx]

Supplementary Table: Bivariate correlation analyses (Pearson) of online-sex use, partner attachment and personality variables (N=2,522)

|  |  |  |  |  |  |  |  |  |
| --- | --- | --- | --- | --- | --- | --- | --- | --- |
| 1. Online-sex use |  | .13*** | .05** | -.19*** | -.08*** | -.02 | .05* | -.01 |
| 1. Anxious attachment |  |  | .23*** | -.16*** | -.04 | .00 | -.12*** | .26*** |
| 1. Avoidant attachment |  |  |  | -.28*** | -.11*** | -.15*** | -.24*** | .23*** |
| 1. (BFI-10) Conscientiousness |  |  |  |  | .16*** | .18*** | .20*** | -.24*** |
| 1. (BFI-10) Agreeableness |  |  |  |  |  | .16*** | .10*** | -.17*** |
| 1. (BFI-10) Openness |  |  |  |  |  |  | .28*** | -.14*** |
| 1. (BFI-10) Extraversion |  |  |  |  |  |  |  | -.34*** |
| 1. (BFI-10) Neuroticism |  |  |  |  |  |  |  |  |

Note: *** p<.001, ** p<.01, * p<.05. BFI-10=Big Five Inventory, 10 item version.
